# Surface-based cortical thickness and gyrification mapping with data-driven prediction of cognitive impairment in prediabetes

**DOI:** 10.1016/j.ibneur.2026.02.013

**Published:** 2026-02-13

**Authors:** Juan Wu, Qi Wang, Feifei Sun, Huiying Liao, Jinhong Song

**Affiliations:** a Department of Radiology, Chengdu Qingbaijiang District People’s Hospital, Chengdu, Sichuan, China; b Department of Radiology, Chengdu Second People's Hospital, Chengdu, Sichuan, China

**Keywords:** Prediabetes, Cognitive impairment, Cortical thickness, Local gyration index, Machine learning

## Abstract

**Background:**

Prediabetes is a serious health condition characterized by blood glucose levels that are higher than normal but not high enough for a diagnosis of type 2 diabetes. It remains unclear whether alterations in cortical morphology occur during the prediabetic stage. This study aimed to investigate changes in cortical thickness and gyrification in individuals with prediabetes, and to explore whether these changes can predict cognitive performance using a machine learning approach.

**Methods:**

T1-weighted MRI scans were acquired from 48 patients with prediabetes and 42 healthy controls. Surface-based morphometric analyses, including cortical thickness and the local gyrification index (LGI), were performed using FreeSurfer. Group comparisons were conducted. Neuropsychological assessments included the Mini-Mental State Examination (MMSE), the Montreal Cognitive Assessment (MoCA), and the Trail Making Test (TMT) Parts A and B. Pearson correlation analyses were conducted to examine associations between morphometric changes and cognitive performance. Furthermore, PyCaret, a machine learning framework, was applied to evaluate the predictive power of cortical features and clinical variables in predicting cognitive performance.

**Results:**

Compared with controls, individuals with prediabetes exhibited significantly reduced cortical thickness in the left inferior temporal gyrus (ITG) and decreased LGI in the left precentral gyrus. TMT-A and TMT-B scores were significantly higher in the prediabetes group, indicating poorer cognitive performance. Cortical thickness in the left ITG was negatively correlated with TMT-B performance (r = −0.54, 95 % CI: −0.71 to −0.31, *p* = 0.0001). Machine learning analysis identified the Extreme Gradient Boosting classifier as the best-performing model (AUC = 0.87, accuracy = 0.80).

**Conclusion:**

Our findings suggest that cortical alterations in the ITG and precentral gyrus are evident during the prediabetic stage and relate to early cognitive dysfunction. These results highlight the potential of combining neuroimaging biomarkers and AI models for early detection and intervention in prediabetes-associated cognitive decline.

## Highlights

• Prediabetes patients had cortical thinning in the left inferior temporal gyrus and reduced gyrification in the left precentral gyrus.
• Individuals with prediabetes exhibited impairments in processing speed and executive function.
• Cortical thickness in the left inferior temporal gyrus negatively correlated with Trail Making Test Part B performance.
• The Gradient Boosting classifier showed promise for early prediction of cognitive impairment in prediabetes populations.

## Introduction

Prediabetes, defined as impaired fasting glucose or impaired glucose tolerance, is an intermediate metabolic condition that precedes type 2 diabetes mellitus (T2DM) (Cai et al., 2020). Its global prevalence is increasing rapidly, with projections indicating that over 470 million people will be affected by 2030 (Makaroff, 2017). Although prediabetes is potentially reversible through lifestyle modification, exercise, and dietary interventions (Zahalka et al., 2024), accumulating evidence suggests that it is associated with an increased risk of stroke, cognitive decline, and Alzheimer’s disease (AD) (Buysschaert et al., 2015). Therefore, early detection and timely intervention are essential.

Nevertheless, the underlying pathological mechanisms of prediabetes-related brain injury remain poorly understood. Previous studies suggest that metabolic and neurological dysfunction may result from central insulin signaling abnormalities, contributing to the pathogenesis of neurodegenerative diseases (Kleinridders et al., 2014). Neuroimaging research has reported structural brain abnormalities in prediabetes, including reduced hippocampal volume and more pronounced global brain atrophy (Dong et al., 2019). Voxel-based morphometry (VBM) studies have identified decreased gray matter volume in regions such as the anterior and posterior cingulate gyri, insula, superior temporal gyrus, and middle temporal gyrus. Furthermore, tract-based spatial statistics (TBSS) studies have demonstrated compromised white matter integrity, particularly in the corpus callosum and bilateral superior longitudinal fasciculus (SLF) (Liang et al., 2019).

Surface-based analysis of cortical thickness and local gyrification index (LGI) provides a sensitive tool for detecting subtle cortical morphological changes. Compared with VBM, surface-based methods achieve vertex-level precision, allowing for the identification of focal gray matter alterations. However, cortical thickness and gyrification changes in prediabetes have not yet been comprehensively studied. Existing literature has predominantly focused on individuals with T2DM, consistently demonstrating cortical thinning in multiple regions, including the bilateral frontal and parietal lobe (Moran et al., 2015) and the sensorimotor cortex (Ferris et al., 2018).

Given that prediabetes is increasingly recognized as a condition associated with early neurocognitive risk, investigating cortical structural changes at this stage may offer a crucial window for early intervention to prevent progressive neurological decline. To date, no study has employed a surface-based, whole-brain approach to compare cortical thickness and LGI between individuals with prediabetes and healthy controls.

This study aimed to: (1) assess cortical thickness and LGI differences between individuals with prediabetes and healthy controls using FreeSurfer; (2) evaluate whether these morphological features are associated with cognitive performance, particularly processing speed and executive function; and (3) employ a PyCaret-based machine learning framework to determine whether morphometric and clinical variables can predict cognitive decline in prediabetes (Xin et al., 2024).

## Methods

### Participants

All participants were retrospectively selected from the Department of Endocrinology, Chengdu Qingbaijiang District People’s Hospital from May 2020 to July 2025. The study protocol was approved by the Ethics Committee of the Chengdu Qingbaijiang District People’s Hospital and written informed consent was waived due to the retrospective nature of this study.

The inclusion criteria for the prediabetic patients were as follows: 1) based on 2014 American Diabetes Association (ADA) diagnostic criteria (Diagnosis and classification of diabetes mellitus, 2013), patients with impaired fasting glucose (IFG) [fasting plasma glucose (FPG) levels 5.6–6.9 mmol/L)], or impaired glucose tolerance (IGT) [2-h values in the oral glucose tolerance test (OGTT) of 7.8–11.0 mmol/L]; 2) Age range 40 from 65 years old. The inclusion criteria for the healthy control group were as follows: 1) OGTT was determined as a normoglycemic person, ie, FPG < 5.6 mmol/L and OGTT 2-hour blood glucose < 7.8 mmol/L; 2) Age range 40–65 years old. Participants were excluded if they had any of the following conditions:1) hypoglycemia, moderate or severe hypertension, psychiatric disease or neurological illness; 2) systemic major organic disease; 3) acceptance of past or present hypoglycemic agents, insulin, and any other treatment that may affect glucose metabolism; 4) contraindications to MRI scan, including metallic implants. Finally, 48 prediabetic patients, 42 healthy controls were enrolled in this study.

### Clinical data

All participants underwent standardized clinical assessments, including a detailed medical interview, physical examination, and laboratory tests. Clinical variables collected included duration of prediabetes, glycosylated hemoglobin (HbA1c), FPG, fasting C-peptide, fasting insulin, triglycerides (TGs), total cholesterol (TC), low-density lipoprotein cholesterol (LDL-C), and high-density lipoprotein cholesterol (HDL-C) (see Table 1).

**Table 1:** Demographic and clinical characteristics of participants.

Variable	Prediabetes	HCs	*p*-value
Age	55.12	55.21	0.9501
Illness duration (y)	2.38	NA	NA
Education (y)	10.94	11.16	0.7106
BMI (kg/m^2^)	24.44	22.9	0.07
Fasting blood glucose (mmol/L)	6.2	4.87	0.006*
2 h after the OGTT (mmol/L)	8.71	5.8	0.00009*
HbA1c (%)	5.94	5.14	0.004*
Cholesterol (mmol/L)	4.91	4.73	0.0137*
Triglyceride (mmol/L)	2.02	1.23	0.0876
High-density lipoprotein (mmol/L)	1.18	1.25	0.0873
Low-density lipoprotein (mmol/L)	2.84	2.76	0.3361
MMSE	28.91	28.79	0.4100
MoCA	27.32	28.17	0.216
TMT-A (seconds)	34.03	33.78	0.7845
TMT-B (seconds)	83.62	77.83	0.0001*

Abbreviations: MMSE, Mini-Mental State Examination; MoCA: Montreal Cognitive Assessment; HC: healthy controls; * *p* < 0.05 indicated statistical significance.

### Neuropsychological assessments

All participants also received neuropsychological assessments. The Mini-Mental State Examination (MMSE) and the Montreal Cognitive Assessment (MoCA) were used to evaluate the general level of cognition (de Boer et al., 2025). Additionally, TMT (Trail Making Test) Parts A and B were used to measure information processing speed and executive function (Bednorz and Religa, 2023).

### MRI data acquisition

Images were acquired using a 3.0 T MR scanner (uMR790, United Imaging Healthcare, Shanghai, China) and a 12-channel head coil. The protocol included the following sequences: T1-weighted images (T1WI) (repetition time [TR]/echo time [TE]/flip angle = 800 ms/205 ms/9°, matrix = 256 × 256, field of view [FOV] = 240 × 240, slice thickness = 3 mm); T2WI (TR/TE/flip angle = 2500/105/150°, matrix = 320 × 230, FOV = 176 × 220 mm, slice thickness = 5 mm); FLAIR (TR/TE/flip angle = 6000 ms/81 ms/90°, matrix = 256 × 198,FOV = 195 × 220 mm, thickness = 5 mm); Each scan was inspected by an experienced radiologist to exclude visible movement artifacts and gross structural lesions.

### Data analysis

Data preprocessing and quantitative metric derivation for all T1WI were performed with FreeSurfer (Fischl, 2012). Within the preprocessing pipeline, a critical first step involved reorienting all images to establish a standardized anatomical position for brain structures. Subsequently, images were resampled to 1 mm isotropic voxels, thereby guaranteeing consistent spatial resolution across the dataset (Fischl and Dale, 2000). Then, the 3DT1 images were registered to Montreal Neurological Institute space, then intensity normalization was performed. To removal of non-brain tissue, automatic skull stripping was conducted. Afterwards, images were inspected for skull stripping errors and segmented into GM, white matter (WM), and cerebrospinal fluid (CSF), following which the cerebral hemispheres were separated. The WM and pial surfaces were obtained by tessellating the WM/GM boundary and deforming the surface by following intensity gradients in order to optimally place WM/GM and GM/CSF boundaries (Dale et al., 1999). Surface inflation and registration to a spherical atlas were performed based on the Desikan et al. (2006), which parcellated the cerebral cortex into 34 regions each hemisphere. The cortical thickness was estimated as the average shortest distance between the pial surface and WM boundary. The LGI was generated using the method of Schaer et al. (2008), which was quantifying the ratio of visible gyral cortex to cortex hidden with the sulci. In order to make a comparison between groups, all the corrected cortical thickness and LGI maps were generated a common average surface and were smoothed using a Gaussian kernel of 15 mm and 10 mm full-width half-maximum respectively, which was prepared for group statistical analysis.

To evaluate whether features derived from cortical architecture can predict cognitive decline in prediabetes at the individual level, we employed an automated machine learning framework, Pycaret (https://pycaret.org/) to construct classification models for distinguishing cognitive decline patients from prediabetes. The Pycaret analysis was described in detail in the Supplementary Materials.

### Statistical analysis

The group differences in the basic demographics, laboratory tests, and psychometric scale scores were assessed using two-sample *t*-test or Mann–Whitney U-test (nonparametric data), and one-way ANOVA. Significance level was set at *p* < 0.05.

We used Freesurfer's Qdec (Query, Design, Estimate, Contrast) general linear model to estimate the differences in the cortical thickness and LGI at each vertex of the surface between the prediabetes and the HC, with age and sex as covariates. Because all images were registered to a common surface template (fsaverage), which inherently accounts for individual differences in brain size, intracranial volume was not included as a covariate (Fischl, 2012). Additionally, education level did not differ significantly between groups (*p* = 0.71); also, education-adjusted cutoff scores according to established norms for the Chinese population were applied (Tan et al., 2015) when assessing cognitive performance, and therefore education was not included as a covariate. Monte Carlo Null-Z Simulation was applied for multiple comparisons (10,000 iterations, cluster-forming *p* < 0.05, clusterwise corrected *p* < 0.05).

To evaluate the relationships between the structural alterations and clinical cognitive scores, we used the command of mris_anatomical_stats in FreeSurfer to compute the average cortical thickness/LGI in the significant clusters; then, the Pearson correlation between the mean cortical thickness/LGI and variables (MoCA, TMT-A, TMT-B) was conducted.

Machine learning model performance was evaluated through several metrics, including accuracy, AUC of the receiver operating characteristic (ROC), recall, precision, F1 score, Kappa, and Matthews correlation coefficient (MCC).

## Results

### General clinical data

Participant demographics and clinical characteristics are presented in Table 1. There were no significant group differences in mean age, education level, gender distribution, BMI, HDL or LDL. However, fasting plasma glucose, 2-hour OGTT glucose, HbA1c, and total cholesterol were significantly elevated in the prediabetes group (*p* < 0.05).

### Neuropsychological scale assessment

Regarding neuropsychological tests, TMT-A and TMT-B scores was significantly increased in prediabetes group, indicative of poorer processing speed and executive function. There were no significant differences between two groups in MMSE and MoCA scores (Table 1).

### Morphological Results

**Fig. 1:**
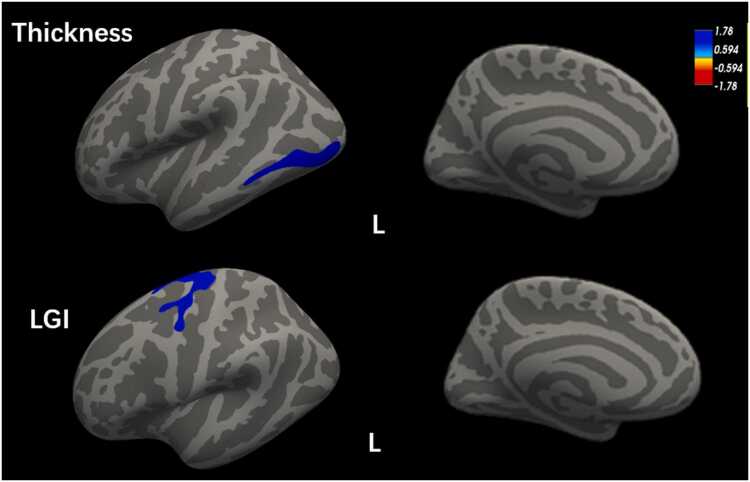
Localization of altered cortical thickness and LGI. The prediabetes patients had reduced cortical thickness (in blue) in the left ITG (the upper panel), and reduced LGI (in blue) in the left precentral gyrus (the lower panel). Abbreviations: ITG, inferior temporal gyrus; LGI, local gyrification index.

Whole‑brain vertex‑wise analysis revealed significant cortical morphological differences. Compared with healthy controls, the prediabetes group exhibited reduced cortical thickness in the left inferior temporal gyrus (ITG) and decreased LGI in the left precentral gyrus. No brain regions showed increased cortical thickness or LGI in the prediabetes group (Fig. 1).

### Associations between morphometrical alterations and cognition

**Fig. 2:**
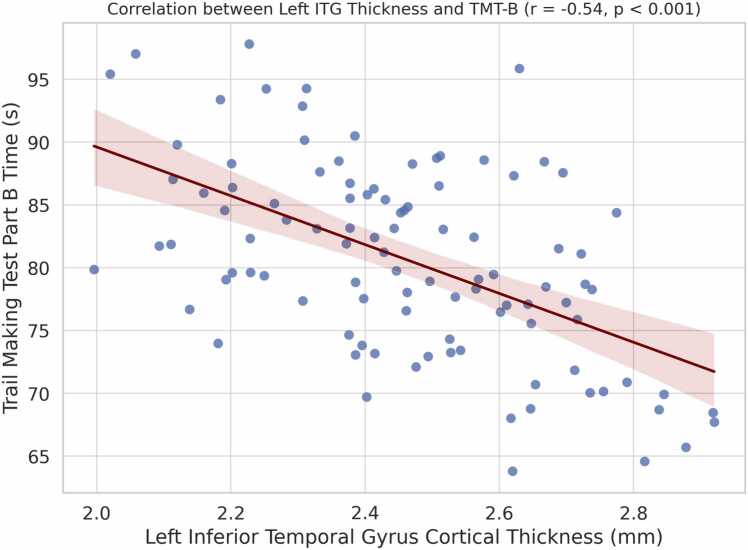
The prediabetic group had a negative correlation between the cortical thickness of the left ITG and the scores of TMT-B, (r = − 0.54, 95 % CI: −0.71 to −0.31, *p* = 0.0001). Abbreviations: ITG, inferior temporal gyrus; TMT-B, Trail Making Test (TMT) Parts B.

Pearson correlation analysis was conducted to assess the relationship between altered morphometrical values (cortical thickness of the left ITG, LGI of the left precentral gyrus) in the prediabetic group and neuropsychological assessments (TMT-A, TMT-B). The correlation coefficient was calculated with 95 % confidence interval using Fisher’s z-transformation. The statistical results showed that the prediabetic group had a negative correlation between the cortical thickness of the left ITG and the scores of TMT-B, (r = − 0.54, 95 % CI: −0.71 to −0.31, *p* = 0.0001) (Fig. 2), suggesting better performance with increased cortical thickness in the left ITG.

### Single‑subject classification of cognitive decline in prediabetes

**Fig. 3:**
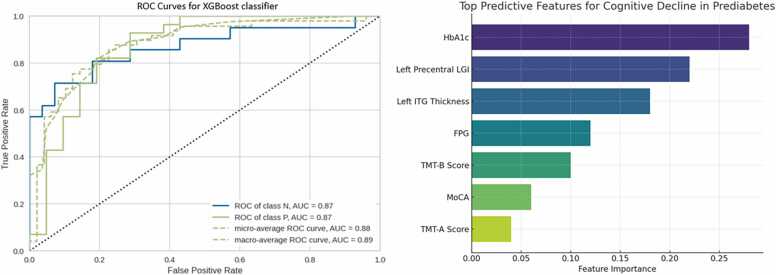
Receiver operating characteristic (ROC) curves for distinguishing early cognitive dysfunction patients from prediabetes and features of the optimal model constructed by the Extreme Gradient Boosting classifier.

Using features derived from cortical morphometric and clinical variables, the Extreme Gradient Boosting classifier (XGBoost) achieved best performance for distinguishing early cognitive dysfunction patients from prediabetes. The model demonstrated an accuracy of 0.80, AUC = 0.87, recall = 0.85, precision = 0.80, F1 = 0.82, and Kappa = 0.57 (Fig. 3).

Feature importance analysis revealed that the strongest predictors included HbA1c, LGI of the left precentral gyrus, and cortical thickness of the left ITG, followed by fasting plasma glucose, TMT‑B scores, and MoCA scores (Fig. 3).

## Discussion

This work aimed to characterize whole-brain cortical morphometric alterations, including changes in thickness and LGI, in prediabetes. By enrolling non-comorbid and never-treated prediabetic patients, we eliminated confounding effects, enabling us to investigate the "pure" neurobiological mechanisms related to prediabetes. Compared to HCs, our research revealed several key findings: prediabetes patients exhibited thinner cortices in the ITG, which positively correlated with cognitive performance. Furthermore, we observed hypogyrification in the left precentral gyrus.

It is well known that diabetes accelerates brain atrophy and cognitive decline in the elderly (Saçmaci et al., 2020; Zhang et al., 2014). In the present study, we found that cognitive dysfunction was present even at the prediabetes stage, particularly in the subdomains of visual and motor processing speed and executive function. Traditionally, cognitive impairment has been attributed to cerebrovascular disease and disruption of frontal subcortical networks. However, our study population included only patients free of cerebrovascular disease. Therefore, our findings indicate that altered cortical morphometry may be associated with early cognitive dysfunction.

A significant finding of this study was the reduced ITG cortical thickness, indicating atrophy of this cortical region in prediabetes patients. This result aligns with a previous VBM study that explored brain volume changes in T2DM patients and revealed decreased gray matter volume in the ITG (Zhang et al., 2019). Additionally, an rs-fMRI meta-analysis focusing on T2DM patients showed reduced resting-state neural activity in the ITG (Xia et al., 2017). Few studies have examined cortical thickness changes specifically in prediabetic populations, and our findings of inferior temporal gyrus thinning contribute to this limited body of literature. This region is hypothesized to be related to changes in glucose metabolism, adiponectin, and insulin resistance, and may be involved in the pathogenesis of prediabetes (García-Casares et al., 2014, García-Casares et al., 2016, García-Casares et al., 2014). Functionally, the ITG is responsible for visual processing and the representation of complex object features, as well as the early recognition of numbers and words (Dien et al., 2013). In the present study, we measured processing speed and executive function using the TMT. The results showed poorer performance in prediabetes patients, and Pearson analysis further revealed that more time spent on the TMT-B test was associated with more severe cortical thickness atrophy in the ITG. The TMT-B is a well-known, reliable, and robust measure of cognitive processing speed. This study provides evidence that cortical thickness atrophy in the ITG may be a potential imaging marker for detecting early cognitive deficits in prediabetes patients.

In addition to exploring cortical thickness, we also conducted a group analysis of gyrification patterns using LGI. The results showed an atypical gyrification pattern in the left precentral gyrus. Literature suggests that the precentral and postcentral gyri are not only associated with sensorimotor function (White et al., 1997), but are also critical for cognitive activities, execution, and attention (Zhang et al., 2021). In turn, the hypogyrification in the precentral gyrus may explain prediabetes patients' poorer performance on the TMT-B test compared to HCs.

The exact mechanism of atypical gyrification pattern is still unclear. According to the theory put forward by van Essen’s (Van Essen, 1997), cortical gyrification pattern primarily mediated by the white matter connectivity (Tang et al., 2021). Thus, we speculated that the observed reduced LGI in precentral gyrus may reflect microstructural abnormalities of the underlying white matter fibers. Indeed, decreased fractional anisotropy in the corpus callosum body was reported in prediabetes patients (Liang et al., 2019). which indicated the white matter structure is damaged and the connectivity of bilateral precentral gyrus is reduced, although limited by its cross-sectional nature, these data raise the possibility that reduced LGI in precentral gyrus may be a sensitive marker of prediabetes pathology.

Moreover, the use of Pycaret-based ML modeling demonstrated that morphometric and clinical features can moderately predict cognitive decline in prediabetes. This data-driven approach highlights the potential of integrating structural MRI and machine learning for personalized risk stratification in prediabetes.

Some limitations of our study should be noted. First, this preliminary study was a single-center study with a relatively small sample size. Although the sample size is relatively limited, several measures were taken to ensure statistical rigor. For morphometric analyses, Monte Carlo simulation with 10,000 iterations was applied for multiple comparison correction. For ML analyses, an independent test set combined with 10-fold cross-validation was employed to minimize overfitting. Importantly, several successful neuroimaging ML studies have demonstrated reliable classification with comparable sample sizes (Chen et al., 2025, Jimenez-Mesa et al., 2024). Nevertheless, future multi-center studies with larger sample sizes are warranted to validate and extend these findings. Second, it should be noted that due to the cross-sectional design of this study, the observed correlations cannot be interpreted as causal relationships. Longitudinal studies are needed to elucidate the temporal relationship between cortical thickness changes and cognitive decline.

In conclusion, this study identified abnormal cortical thickness and gyrification patterns in patients with prediabetes. The results suggest that ITG and precentral gyrus may be involved in the pathogenesis of prediabetes-related brain injury, which indicates new directions for early intervention and prevention of cognitive impairment in prediabetic patients.

## CRediT authorship contribution statement

**Juan Wu:** Writing – original draft, Visualization, Validation, Software, Resources, Methodology, Investigation, Data curation, Conceptualization. **Huiying Liao:** Writing – original draft, Validation, Software, Data curation, Conceptualization. **Jinhong Song:** Writing – review & editing, Writing – original draft, Supervision. **Qi Wang:** Visualization, Validation, Investigation, Data curation, Conceptualization. **Feifei Sun:** Methodology, Data curation.

## Ethical approval

The study was approved by the Ethics Committee of Chengdu Qingbaijiang District People’s Hospital.

## Consent

Written informed consent was waived due to the retrospective nature of this study, as all MRI scans were performed for clinical purposes and the data were retrospectively selected from clinical cases that met our study criteria.

## Funds statement

The Natural Science Foundation of Sichuan Province (2025ZNSFSC1769).

## Declaration of Competing Interest

The authors declare that they have no known competing financial interests or personal relationships that could have appeared to influence the work reported in this paper.

## Data Availability

The datasets produced during this study can be obtained upon request directed to the corresponding author.

## Footnotes

Appendix A Supplementary data associated with this article can be found in the online version at doi:10.1016/j.ibneur.2026.02.013.
