# Supplementary material for: Surface-based cortical thickness and gyrification mapping with data-driven prediction of cognitive impairment in prediabetes
Source: IBRO Neurosci Rep. 2026 Feb 13;20:299–304. doi: 10.1016/j.ibneur.2026.02.013 (PMC12927054; doi:10.1016/j.ibneur.2026.02.013)
Supplement: Supplementary file 1 — Supplementary material [file mmc1.docx]

**Surface-Based Cortical Thickness and Gyrification Mapping with Data-Driven Prediction of Cognitive Impairment** **in Prediabetes**

**Supplementary Material**

**Supplementary Material**

**Methods**

*Cortical feature extraction*

The mean value of cortical thickness and LGI were extracted based on the Desikan-Killiany atlas employing the 'aparcstats2table' command within the Freesurfer. Totally, 136 cortical features were computed for each patient (68 cortical thickness, 68 cortical LGI).

*Cognitive impairment individual in prediabetes prediction models created by Pycaret*

Pycaret is a Python wrapper for a number of machine learning (ML) frameworks and packages (including scikit-learn, XGBoost, and LightGBM), with the purpose of hyperparameter tuning and assembling techniques to improve the efficiency of the recognized model. It can speed up the process of creating the best ML model, enabling researchers to carry out experiments rapidly and effectively in low-code environment. Cognitive decline in the prediabetes group was defined as meeting either of the following criteria: (1) MoCA score < 26 (established screening threshold for mild cognitive impairment; Nasreddine et al., 2005), or (2) TMT-B completion time exceeding 1.5 standard deviations above the healthy control group mean (i.e., > 95.8 seconds, calculated as 77.83 + 1.5 × 12). This combined criterion was chosen to capture both global cognitive impairment and domain-specific executive dysfunction. Based on these criteria, 17 participants (35.4%) were classified as having cognitive decline and 31 (64.6%) as cognitively normal. In this study, we use the classification module in Pycaret: this is a supervised ML module for binary classification of elements based on 15 ML algorithms. To construct prediction models, the data set comprised of cortical thickness, LGI, and Clinical variables (duration of prediabetes, HbA1c, FPG, fasting C-peptide, fasting insulin, TGs, TC, LDL-C, and HDL-C) were fed separately into Pycaret. By default, Pycaret divides each set into a training cohort (70%) and an independent test cohort (30%). To prevent data leakage, the dataset was first split into training and test cohorts before any preprocessing or model training. The test cohort was held out and only used for final model evaluation. Each feature set was trained on 15 ML models and the stability of models was evaluated by performing 10-fold cross-validation, the performance of each model and the radiomics features that contributed most being automatedly generated on the training cohort. The top 5 most accurate models were then and subjected to hyperparameter tuning, and the tuned models ensembled using the blending method. The models were assembled and evaluated on the test cohort. Finally, the performance parameters were obtained.
